# Effects of a Smartphone-Based, Multisession Interpretation-Bias Modification for Anxiety: Positive Intervention Effects and Low Attrition

**DOI:** 10.3390/ijerph20032270

**Published:** 2023-01-27

**Authors:** Delhii Hoid, Dong-Ni Pan, Chun Liao, Xuebing Li

**Affiliations:** 1 Key Laboratory of Mental Health, Institute of Psychology, Chinese Academy of Sciences, Beijing 100101, China; 2 Department of Psychology, University of Chinese Academy of Sciences, Beijing 100049, China

**Keywords:** anxiety, negative interpretation bias, smartphone-based interpretation-bias modification, positive intervention effects, low attrition

## Abstract

While interpretation-bias modification (IBM) is an effective intervention for treating anxiety, it is not broadly used in clinical or daily practice. To this end, this study developed and tested a smartphone-based IBM application. We adopted the ambiguous situation paradigm as an intervention task in conjunction with robust training materials that broadly covered situations encountered in daily life. We recruited participants with high-trait anxiety and divided them into three groups: (1) positive training; (2) 50% positive–50% negative training; and (3) no-training control. The first two groups completed 28 days of smartphone-based training (IBM in positive cases), and all groups completed six rounds of assessments. The smartphone-based IBM training changed positive and negative endorsements and more specific measures of interpretation bias, thus reducing anxiety. The results also showed that changes in the number of negative interpretations played a mediating role in anxiety reduction. It is notable that the attrition rate was extremely low across the experiment. Our follow-up showed that positive gains persisted throughout the intervening period. Smartphone-based IBM can help individuals with anxiety shift negative biases, broaden their thoughts, enhance their information processing, and effectively target the clinical features of anxiety.

## 1. Introduction

Anxiety disorder is a severe mental illness with a high prevalence rate [1]. Individuals thus affected may experience constant fear, worry, and even somatic symptoms [2], all of which can make it difficult to complete tasks. In turn, the condition leads to a variety of economic and social health care burdens [2]. These problems are compounded by the fact that nearly 90% of anxious individuals do not receive adequate treatment, often due to financial barriers, stigmas, and insufficient access to care [3].

Using the cognitive theory of anxiety as a framework, studies have shown that negative interpretation biases causally contribute to anxiety and its symptoms [4,5]. In accordance with this, the treatment known as interpretation bias modification (IBM) has successfully been used to relieve anxiety symptoms by modifying negative biases in affected individuals [6,7]. While IBM has also been praised for its convenience and low cost, it is not widely implemented due to technical barriers. To promote the use of this method in anxiety relief, this study developed and tested a smartphone-based IBM training application (app), focusing on intervention effects for negative interpretation bias and anxiety. In turn, we aimed to clarify critical factors in the continued development of mobile health (mHealth)-based interventions at large. We believe such efforts will ultimately work to popularise the practical application of psychotherapy approaches that can prevent severe mental disorders.

As previously mentioned, IBM targets negative styles of interpretation. Typically, each trial in the modification procedure begins with a piece of ambiguous information; presented as such, participants are asked to make a decision that should be facilitated by a particular interpretation, and then to conclude on the meaning of this information. This repetition of the decision-making process is expected to induce different interpretation biases. Previous studies have shown that these modification procedures are effective. For example, in Mathews and Mackintosh [8], unselected participants read a short description of an ambiguous social situation that ended with a word fragment requiring their resolution; specifically, the constructed word fragments were designed to result in either a benign or threatening ending, thereby inducing different emotional interpretation biases. After many trials, the results showed a remarkable induction of positive or negative interpretation biases, reflected by the assignment of respective induction conditions. In another study, Amir, et al. [9] used the Interpretation Modification Program (IMP) to alter interpretation biases in socially anxious participants, thus reducing their symptoms. An internet-based, multi-session IBM study further indicated that the beneficial effects of the IBM program for SAD patients were still stable over a six-week follow-up period [10]. Advancing this concept, many researchers were not satisfied with simply replicating the results of existing IBM studies, and instead focused on broader uses and potential far-transfer effects. In one case, Wilson, MacLeod, Mathews and Rutherford [7] tested the ecological validity of interpretation modification, thus finding that such modifications could positively affect anxiety vulnerability. Moreover, some studies expanded on the applicability of IBM by using it as a cognitive intervention to treat other issues, including obsessive-compulsive disorder and substance abuse behaviour [11,12].

Despite its known effectiveness in reducing anxiety, most laboratory and web-based IBM approaches are unsuitable for daily treatment because of time and space limitations. On the other hand, mHealth technologies have catalysed new and promising psychotherapy approaches [13]. For example, Carl, et al. [14] developed the Daylight programme, a novel, smartphone-based cognitive behavioural therapy (CBT) intervention to help participants learn and apply relevant concepts and skills. According to their results, Daylight can reduce the effects of generalised anxiety disorder and various secondary symptoms, including worry and sleep difficulty. Purkayastha, et al. [15] also pointed out that smartphone-based CBT (or interventions) are more accessible and cost-effective than their web-based counterparts. Despite this potential, no previous studies have used mobile devices to promote IBM for reducing anxiety [16,17]. It is also worth noting that only two studies even examined the feasibility of smartphone-based IBM [16,18]. In both cases, participants accessed IBM training through website links they received on their mobile phones, with no differences to previous, laboratory-based or online training studies; moreover, each study implemented homogenous training and assessment tasks. Thus, it is impossible to conclude whether either of these handheld IBM training programmes could broadly change interpretation biases in daily ambiguous situations. To address this gap in the literature, this study developed and tested a highly accessible, smartphone-based IBM application designed to help individuals with anxiety. Accordingly, our primary goal was to evaluate the effects of the novel app regarding the modification of interpretation bias and the reduction of anxiety.

A meta-analysis systematically reviewed features that could increase engagement with mental health apps, concluding that it was essential to facilitate the app’s primary purpose: clinical or intervention effects [19]. Several preliminary studies found that changes in interpretation biases play a critical role in anxiety reduction. For example, Salemink, et al. [20] calculated interpretation bias indices for participants by subtracting their mean scores on negative interpretation from those on positive interpretation, thus concluding that altered interpretations mediated changes in anxiety. Beard and Amir [21] asked socially anxious individuals to complete eight rounds of IMP training, thereby demonstrating that changes to benign interpretation biases could mediate the modification’s effects on anxiety. By contrast, Sun, Yang, Zhang, Xiao and Cui [18] reported that an altered interpretation bias did not mediate anxiety changes. One possible explanation for these inconsistent results is that interpretation biases were measured differently between studies, which may imply that the central issue for improving IBM’s effectiveness is using more specific measures for interpretation biases and testing their influences on anxiety.

Therefore, the present study focused on three specific measures of negative interpretation bias: namely, the number participants made, their ranks, and relevant beliefs. This offers a vivid picture of how individuals process ambiguous information related to daily life [22]. According to Beck and Clark [23], the primal threat appraisal can block constructive thoughts in individuals with anxiety, who may therefore continuously interpret ambiguous information as threatening. Further, the number of negative interpretations an individual makes is directly related to recalls of how they previously interpreted similar ambiguous situations, which can result in a memory bias [24]. Thus, a higher number of negative interpretations reflects a narrower and more inflexible view. Another important aspect is the rank of a negative interpretation, as this represents the priority or speed of its processing; here, high-priority interpretations can result in close associations between the stressful outside world and negative biases, thus maximising their adverse effects on anxiety. Finally, individuals activate an imagination process when attempting to solve ambiguity. In this context, anxious individuals are prone to an intrusion of negative self-imageries and thoughts [25], thus inducing a belief in negative interpretations. As reported in the previous studies, this is a clinical feature of anxiety disorder that may require intervention [26,27]. In total, the number and rank of negative interpretations and beliefs are specific embodiments of interpretation biases in daily life. However, we know of no previous studies that have investigated how IBM influences these dimensions and the subsequent influence on anxiety reduction. Thus, our second study goal was to explore how IBM affected these crucial measures of interpretation bias and their subsequent influences on the intervention.

In summary, this study developed and tested a smartphone-based IBM application with the aim of evaluating the potential of broad mobile use. As such, we recruited a sample of individuals with high-trait anxiety to complete different IBM training components based on the smartphone application for 28 days. In addition to the positive IBM training, an application with active control training was developed to test the placebo effects of IBM [16]. Each participant was subjected to multiple assessments on their interpretation bias and anxiety level. Following the experimental period and post-test, we also conducted a one-month follow-up assessment to test the longevity of intervention effects. Finally, the present study will examine the mechanism of IBM on anxiety reduction using multi-dimensional measures of interpretation bias and longitudinal data. For the experimental results, two hypotheses were formulated.

**H1.**  *Based on the positive effects of multi-session IBM and its long-lasting impact [3,10], we hypothesize that the smartphone-based IBM app will effectively change negative interpretation biases and reduce anxiety. These positive results will remain at the time of the one-month follow-up.*

**H2.**  *Despite the inconsistent results regarding the mediating roles of the interpretation bias, the specific measures of negative interpretation bias used in the present study better reflect the clinical features of anxiety. Thus, we expect that changes in interpretation biases would be a crucial premise for reducing anxiety. Moreover, the number, the priority, and the belief about negative interpretation will play mediating roles in anxiety reduction.*

## 2. Materials and Methods

### 2.1. Participants

Initially, 486 Chinese undergraduate students participated in our Internet screening process, wherein they completed the trait anxiety subscale of the Chinese version of the State-Trait Anxiety Inventory (STAI) [28,29]. To create a study sample, we selected 100 students with scores above 50 in trait anxiety [30] and randomly divided them into the positive training; 50% positive–50% negative training (active control condition, hereafter 50-50); and no-training control (NTC) groups. However, six participants dropped out during the training period and were thus excluded from our analysis. As such, our final study sample included 94 participants who completed the entire study course (positive: *n* = 34; 50-50: *n* = 31; NTC: *n* = 29). According to a previous meta-analysis [31], the effect size (Cohen’s f) of pre–post training changes in a positive interpretation bias is 0.215. Based on this, we needed at least 18 participants in each group to achieve a statistical power of 95%; we thus met this requirement. Three groups were matched for their age, gender, years of education, and anxiety scores (Table 1). All participants provided written informed consent before the experiment was conducted.

### 2.2. Materials

#### 2.2.1. Intervention Task Stimuli

The IBM training stimuli were first set. For the modification training component, we selected 113 ambiguous scenarios adapted from Mathews and Mackintosh [8]. We also conducted an accuracy check in which 250 individuals who were not involved in our interventions rated whether the scenarios could truly happen in daily life. Scenarios were evaluated using a 5-point scale ranging from 1 to 5, with higher scores reflecting better representations. After excluding six ambiguous scenarios with average scores below 3, the training materials consisted of 107 ambiguous scenarios with a mean rating of 3.9. Of all stimuli, 70% were related to the social context (e.g., giving a presentation in public), while 30% pertained to self-relevant or other threats (e.g., threats to physical health). Each associated word fragment was designed to induce one of two biases; that is, either a benign interpretation bias, thus making the situation positively understood by solving it, or a threatening interpretation bias, thus making the situation negatively understood. All such fragments were constructed so that only one solution was available.

We developed the smartphone-based IBM training programme and WSAP assessment task via Android studio for Windows using JAVA and Kotlin. Accordingly, the current versions of each are only supported on the Android system (versions should be newer than 4.1). We generated the training and assessment data in a format similar to E-data that could be exported and pre-processed in Microsoft Excel. No technical issues emerged during the test or training phases. Recently, we translated the IBM training and assessment tasks via the WeChat Mini Program (Tencent), which is an online platform that is free of the limitations of operational systems and can thus benefit the broader population. This also enhanced flexibility when modifying and updating the intervention tasks and materials for different groups.

#### 2.2.2. Smartphone-Based Intervention Task

**Figure 1:**
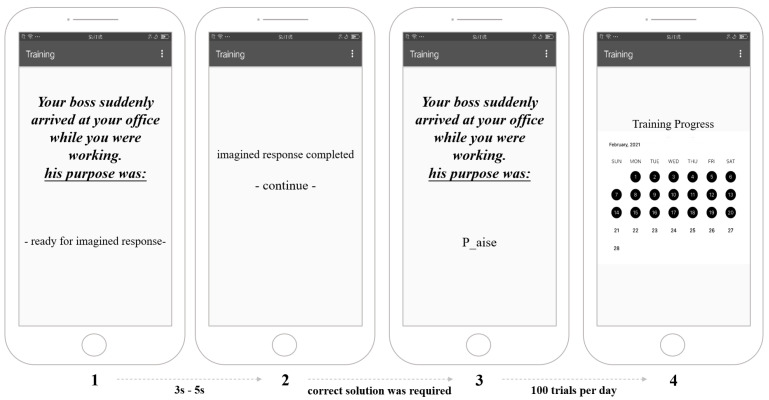
Smartphone-based interpretation-bias modification training.

The task began with a brief description of an ambiguous situation that participants were asked to read while imagining that they were the person therein involved. They were then presented with a word fragment that they needed to complete to determine the meaning of the situation (Figure 1). In the positive condition, all word fragments were designed to induce benign interpretation biases. In the 50-50 condition, equal percentages of all word fragments were designed to induce benign and threatening interpretation biases. Each day, the smartphone-based IBM task contained 50 randomly selected trials from our database. Each participant was required to complete a full course of the task twice daily (100 total trials per day, approximately 12 to 15 min for all 100 trials).

#### 2.2.3. Interpretation Bias Measures

**Figure 2:**
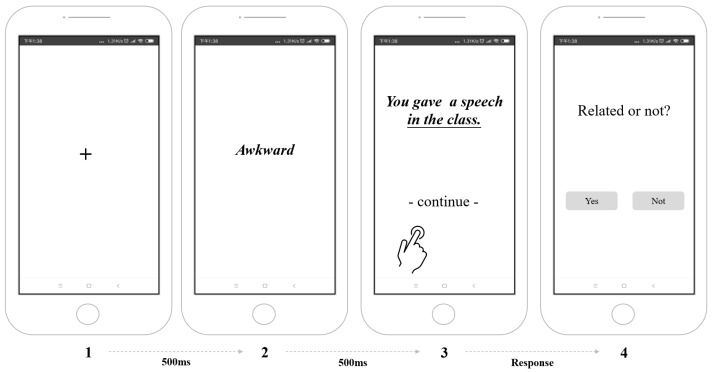
Smartphone-based WSAP task.

Here, participants completed WSAP tasks. Each trial began with a fixation at the centre of the smartphone screen for 500 ms, followed by a word with either a benign (e.g., “stirring”) or threatening (e.g., “awkward”) meaning for 500 ms. Then, an ambiguous sentence (e.g., “You gave a speech in the class”) appeared on the screen; this remained until participants activated the touchscreen, indicating that they had read the sentence. Finally, they were required to indicate whether the word and sentence were related by pressing a “Related or unrelated?” button on the screen. A subsequent trial initiated after such a response. Figure 2 illustrates the WSAP procedure.

Our bank of interpretation questionnaires included 40 scenarios, collected from the Ambiguous Social Situations Interpretation Questionnaire [22], Interpretation and Judgmental Questionnaire [32], and the interpretation questionnaire used by Butler and Mathews [33]. These scenarios constituted four interpretation questionnaires in which each scenario was presented twice. Each questionnaire contained 20 ambiguous situations. Two of the four questionnaires were used twice because each participant needed to complete six assessments.

In the interpretation questionnaires, each ambiguous situation (e.g., the audience clapped when you finished your speech) was followed with the open-ended question “why?” Participants wrote any explanation that came to mind after reading the description of the situation, then turned the page and ranked three predetermined alternative explanations according to which was most likely to enter their mind. Specifically, each set of explanations included three different interpretations; that is, benign (“You made a stirring speech”), neutral (“Basic social etiquette”), and threatening (“The audiences find your speech uninteresting, they thought it is finally over”). Finally, they rated whether each of the three alternative explanations was likely to occur on a 9-point scale ranging from 1 to 9.

We classified the open-ended answers by referring to Stopa and Clark [22]. Here, two graduate students were designated as raters. The first rater independently classified all data from the six assessment rounds, while the second rater independently classified 30 datapoints from each round. Based on our analysis, the mean inter-rater agreement was satisfactory (0.94 for benign interpretations, 0.88 for neutral interpretations, and 0.91 for threatening interpretations). Finally, the number of negative interpretations was recorded, with averages calculated for their ranks and beliefs.

#### 2.2.4. Anxiety Level Measures

We collected self-reported data on state and trait anxiety using the Chinese version of the 40-item State-Trait Anxiety Inventory [28,29]. Items 1–20 measured state anxiety, while items 21–40 measured trait anxiety; each of which were answered on a 4-point scale. The Chinese version of the STAI is valid for measuring both aspects based on Cronbach’s α values of 0.88 and 0.91 [34]. Moreover, the inventory’s convergent validity has been confirmed based on significant correlations with other measures of psychological well-being [28].

### 2.3. Procedure

**Figure 3:**
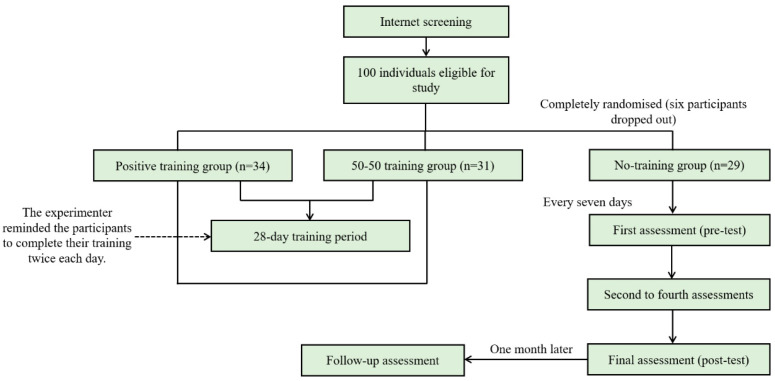
Flowchart showing the experimental study procedure.

Prior to commencement, this study was approved by the Institutional Review Board affiliated with the Institute of Psychology, Chinese Academy of Sciences [IPCAS2004]. After participant recruitment, eligible participants were randomly assigned to different groups by drawing lots. The experiment with a single-blind method lasted 28 days, during which individuals in both interpretation-modification and interpretation-control conditions received training twice daily. The experimenter reminded the participants to complete the training at 11:30 am and 6:00 pm each day over the training period. Accordingly, the experimenter received feedback and kept their completion records. None of the 94 analysed participants missed training days. In addition to the pre- and post-test assessments, all participants in the three study groups completed assessments every seven days. All three groups completed follow-up assessments one month after the post-test. Figure 3 shows a flowchart of the experimental study procedure. Regarding compensation, those in the training groups received RMB 180 each, while those in the NTC group received RMB 100 each. After the experiment, the 50-50 NTC groups were asked to complete at least two weeks of positive training to avoid possible adverse effects.

### 2.4. Statistical Analysis Plan

The repeated measures ANOVA was our main method for observing interpretation bias and anxiety changes. More specifically, we conducted a 2 (interpretation endorsement: benign/threatening) × 3 (groups: the positive training/50-50 training/NTC) × 5 (time: pre-test/2/3/4/post-test) repeated measures ANOVA for the WSAP task, and a 3 (groups: the positive training/50-50 training/NTC) × 5 (time: pre-test/2/3/4/post-test) repeated measures ANOVA for the interpretation questionnaire and anxiety scores. We used the Bonferroni correction to post-hoc test the main effect, and the partial eta squared was used to indicate the ANOVA effect size. When an interaction was significant, we conducted a simple effects test to reveal changes or differences in the variables (simple simple effects for interactions involving three factors). We conducted our statistical analyses using Jamovi 1.6.8.0 [35], with significance established at 0.05.

We conducted a multiple mediation analysis using the bootstrapping method with 5000 samplings and a 95% confidence interval. The predictor was the group assignment, in which the positive training, 50-50 training, and NTC groups were coded as 1 to 3, as Ji, Baee, Zhang, Calicho-Mamani and Teachman [3] reported that the 50-50 training condition might influence the outcomes of the intervention as an alternative active condition. The measures of interpretation bias at the third assessment were considered mediators, while the dependent variable was the post-test anxiety score. We conducted the mediation analysis using SPSS 26.0 (IBM Corp., New York, NY, USA) and PROCESS version 3.4.1.

## 3. Results

### 3.1. Changes in Interpretation Bias

#### 3.1.1. WSAP Task

The main effects of interpretation endorsement (*F* (1, 91) = 101.79, *p* < 0.001, *η*_p_^2^ = 0.528), group (*F* (2, 91) = 4.87, *p* = 0.01, *η*_p_^2^ = 0.097), and time (*F* (4, 364) = 19.5, *p* < 0.001, *η*_p_^2^ = 0.176) were significant. The interactions between interpretation endorsement and group (*F* (2, 91) = 13.98, *p* < 0.001, *η*_p_^2^ = 0.235), group and time (*F* (8, 364) = 2.74, *p* = 0.012, *η*_p_^2^ = 0.057), and interpretation endorsement, group, and time (*F* (8, 364) = 8.53, *p* < 0.001, *η*_p_^2^ = 0.158) were also significant. Regarding the three-factors’ interaction, further tests revealed that the benign interpretation endorsement of the positive training group enhanced significantly at the second assessment (*p* = 0.015) and was finally enhanced above 20% at the final assessment (*p* < 0.001; compared with the first assessment). However, the other two groups failed to show such change during all five assessments. In addition, the benign interpretation endorsement of the positive training group was significantly higher than that of the NTC group since the third assessment (*p*s < 0.001).

**Figure 4:**
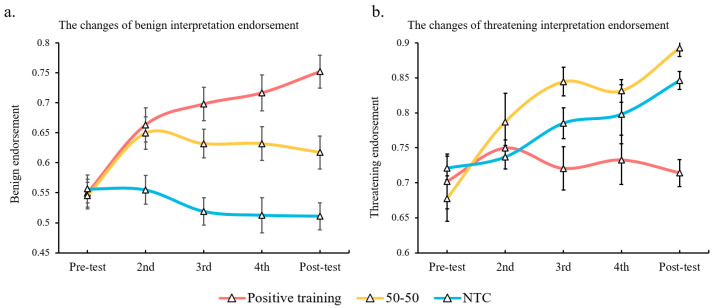
Changes of benign (**a**) and threatening (**b**) interpretation endorsements for three groups during the experiment.

For the threatening interpretation endorsement, when compared to the first assessment, the 50-50 group and the NTC group finally increased by 22% (*p* < 0.001) and 13% (*p* = 0.011), respectively, while the positive training group did not display such an increase. Additionally, at the final assessment, the threatening interpretation endorsement of the 50-50 group—but not the NTC group—became significantly higher than that of the positive training group (mean difference = 18%, *p* < 0.001). For the results of the WSAP task, see Figure 4.

#### 3.1.2. Interpretation Questionnaire

The number of negative interpretations. The main effects of time (*F* (4, 364) = 6.2, *p* < 0.001, *η*_p_^2^ = 0.064) and group (*F* (2, 91) = 15.3, *p* < 0.001, *η*_p_^2^ = 0.251) were significant. Post-hoc tests revealed that participants made fewer negative interpretations after the fourth and fifth assessments (*p* = 0.019 and *p* = 0.004, respectively; compared to the first assessment). Additionally, the positive training group made fewer negative interpretations than the other two groups made (*p*s < 0.001). A significant interaction between time and group (*F* (8, 364) = 2.84, *p* = 0.005, *η*_p_^2^ = 0.059) was observed. Further tests revealed that for the positive training group, when compared with the first assessment, the number of negative interpretations had reduced significantly (*p* = 0.009) from the third assessment and was down to an average of 4.75 at the final assessment (*p* < 0.001). For the other two groups, however, the numbers did not change significantly during the five assessments. The positive training group finally made an average of 3.9 fewer negative interpretations than the 50-50 group (*p* = 0.004) and an average of 3.6 fewer than the NTC group (*p* = 0.014).

Regarding the ranks of the negative interpretations, significant main effects of time (*F* (4, 364) = 18.54, *p* < 0.001, *η*_p_^2^ = 0.169) and group (*F* (2, 91) = 8.42, *p* < 0.001, *η*_p_^2^ = 0.156) were found. Post-hoc tests indicated that participants ranked the negative interpretation significantly lower since the third assessment (*p* < 0.001), and the positive training group ranked negative interpretations significantly lower than the other two groups did (*p* = 0.002 and *p* = 0.003, respectively). A significant interaction between group and time (*F* (8, 364) = 4.91, *p* < 0.001, *η*_p_^2^ = 0.097) was demonstrated. Further tests indicated that, when compared with the first assessment, the positive training group ranked the negative interpretations significantly lower since the third assessment (*p* < 0.001). However, the other two groups did not exhibit such a significant change.

Regarding the belief about negative interpretations, significant main effects of time (*F* (4, 364) = 3.77, *p* = 0.005, *η*_p_^2^ = 0.04) and group (*F* (2, 91) = 5.52, *p* = 0.005, *η*_p_^2^ = 0.108) were shown. Post-hoc tests indicated the negative beliefs of the third assessment were significantly weaker than those of the first and second assessments (*p* = 0.023 and *p* = 0.01, respectively). The interaction between time and group was significant (*F* (8, 364) = 5.42, *p* < 0.001, *η*_p_^2^ = 0.106), whereby the beliefs with respect to negative interpretation from the positive training group became significantly weaker than that of the 50-50 group (*p* = 0.032) since the third assessment.

**Figure 5:**
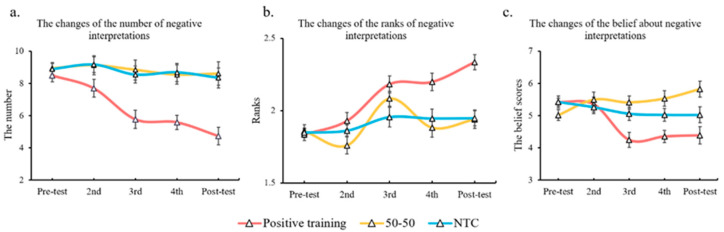
Changes in number (**a**), rank (**b**), and belief (**c**) about negative interpretations for three groups during the experiment.

Results for changes in number, rank, and belief are shown in Figure 5.

### 3.2. Anxiety

With respect to the state anxiety of participants, the main effect of time (*F* (4, 364) = 7.36, *p* < 0.001, *η*_p_^2^ = 0.075) and the interaction between time and group (*F* (8, 364) = 3.12, *p* = 0.002, *η*_p_^2^ = 0. 064) were significant. Post-hoc tests found that anxiety scores at the fourth and fifth assessments were significantly lower than the scores from the pre-test (*p*s < 0.001). A further simple effect test revealed that the trait anxiety scores in the positive training group reduced significantly at the fourth assessment (*p* < 0.001; compared with pre-test).

**Figure 6:**
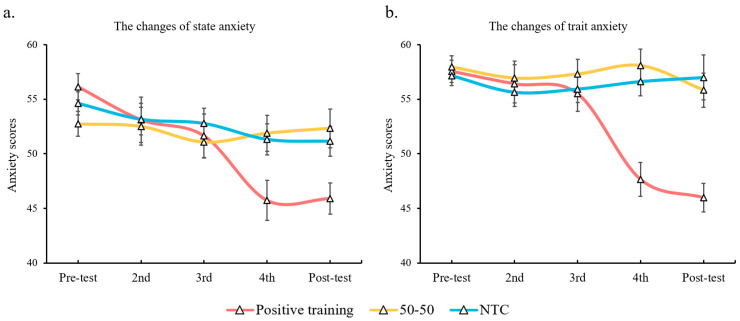
Changes of state (**a**) and trait (**b**) anxiety for three groups during the experiment.

With respect to the trait anxiety of participants, the main effects of time (*F* (4, 364) = 8.08, *p* < 0.001, *η*_p_^2^ = 0.082) and group (*F* (2, 91) = 5.71, *p* = 0.005, *η*_p_^2^ = 0.112) and the interaction between time and group (*F* (8, 364) = 7.98, *p* < 0.001, *η*_p_^2^ = 0.149) were significant. When compared with the first assessment, the trait anxiety scores were reduced significantly at the fourth and fifth assessments (*p*s < 0.001). The positive training group became less anxious than the other two groups (*p* = 0.007 and *p* = 0.035, respectively). A further simple effect test revealed that the trait anxiety scores in the positive training group were reduced significantly at the fourth assessment (*p* < 0.001; compared with all the former assessments) and that they were significantly lower than the scores in the other two groups at the same assessment (*p* < 0.001 and *p* = 0.005, respectively). At the fifth assessment, the trait anxiety scores in the positive training group were still significantly lower than those in the other two groups (*p* = 0.005 and *p* = 0.001, respectively). For the changes in anxiety for all groups, see Figure 6.

### 3.3. Follow-Up Assessment

#### 3.3.1. Changes in Interpretation Bias

Neither the main effects of time nor the interactions between time and group were significant. Main effects of the group were found for each measure of interpretation bias. Post-hoc tests showed that the positive changes in the interpretation bias remained at the one-month follow-up in the positive training group. For the results of the repeated measures ANOVAs, see Table 2.

#### 3.3.2. Anxiety

For different measures of anxious feelings, the interactions between time and group were non-significant (state: *p* = 0.619; trait: *p* = 0.704). For state anxiety, the significant main effects of time (*F* (1, 91) = 5.002, *p* = 0.028, *η*_p_^2^ = 0.052) and group (*F* (2, 91) = 12, *p* < 0.001, *η*_p_^2^ = 0.209) were observed. Post-hoc tests revealed that anxiety scores at the follow-up assessment were significantly higher than those at post-test (*p* = 0.028). Additionally, the state anxiety scores in the positive training group were lower than those of the other two groups (*p*s < 0.001). Similarly, the main effect of group was significant for trait anxiety, and the positive training group showed a significantly lower anxiety level than other groups (*p*s < 0.001). These results indicated that reduced anxiety scores persisted in the positive training group, even without the modification for a month.

### 3.4. The Mediation Analysis

The results indicated that only the number of negative interpretations could play the role of the meditator. Specifically, the group assignment significantly predicted the number of negative interpretations (*β* = 1.655, *SE* = 0.519, *t* = 3.187, *p* = 0.002). The number of negative interpretations significantly predicted the change in anxiety (*β* = 0.484, *SE* = 1.502, *t* = 3.125, *p* < 0.001). The total effect (*c* = 5.63, *SE* = 1.2, *p* < 0.001) and the direct effect (*c’* = 4.695, *SE* = 1.502, *p* = 0.002) of the group assignment on anxiety was significant, and the indirect effect through the number of negative interpretations on anxiety was also significant (*β* = 0.802, *SE* = 0.481, CI: [0.039, 1.9], see Table 3).

## 4. Discussion

In this study, we developed and tested a smartphone-based IBM application with a focus on its effects on interpretation bias and anxiety. In brief, our results suggested that the smartphone-based IBM app is feasible and effective for broad use in daily life. After 28 days of IBM training, the positive training group made more positive endorsements during the WSAP task, according to our within- and between-group comparisons. Moreover, this group did not show increased negative endorsements, as was found in the other study groups. Looking at the specific measures of interpretation bias, the positive group also exhibited positive changes in their interpretation biases. In summary, these findings showed that our smartphone-based IBM training programme effectively reduced anxiety and may have important clinical implications, especially based on our one-month follow-up assessment, which showed that the positive group had retained the aforementioned benefits. Further, our mediation analysis indicated that changes in the number of negative interpretations played a crucial role in anxiety reduction, which should enlighten future explorations.

In addition, our smartphone-based IBM app positively influenced different aspects of the interpretation bias. First, although the training and assessment tasks were not homogeneous, the AS-paradigm-based IBM increased positive endorsements during the WSAP task within a short period of time (two weeks), thus indicating that daily use can enhance the positive transfer effects. We also found that the smartphone-based IBM could protect individuals from making extremely negative endorsements. Second, our app positively changed interpretation biases, reflecting a more detailed interpretation process. According to the baseline measurements, anxious individuals made more negative interpretations. This supports Beck and Clark [23], who reported that individuals with an anxiety disorder tended to be inflexible and remain trapped in narrow, threatening thoughts when facing anxiety-eliciting situations. To address this, our training materials covered a variety of daily events in which modification could provide many possible positive outcomes for anxious individuals to absorb, regardless of whether they agreed with the suggested methods for resolving ambiguities. This evidence shows that anxious individuals can flexibly reappraise different situations after engaging in IBM, thus preventing negative biases from impeding their progress. Moreover, the number of negative interpretations significantly decreased. Previous studies demonstrated that IBM can influence memory bias [24,36]. An fMRI study also found greater memory-retrieval-related brain activation during positive IBM, which indicated that participants recalled possible positive interpretations [37] rather than negative ones. In conjunction with the results of our mediation analysis, these findings suggest that individuals who accept the IBM training may recall fewer negative experiences when facing ambiguity, which may serve as the premise for reducing anxiety. If an individual makes many benign interpretations but refuses to take them seriously, then they will minimise the positive effects of those interpretations. In this regard, the IBM training requires participants to repeatedly resolve constructed word fragments positively, which may reduce the possibility that they will first consider negative interpretations when facing ambiguous situations. Thus, the positive training group gradually placed a lower priority on negative interpretations. According to the literature, this is an important area of remediation. For example, Muris and Field [5] found that individuals with anxiety disorder readily attached threatening meanings during the interpretation stage. At the same time, Hallion and Ruscio [38] reported that negative cognitive biases were only activated under stressful situations. In summary, it is reasonable to assume that IBM training can influence the order or priority individuals give to different interpretations and perhaps eliminate close associations between stressful external factors and negative interpretations. Loscalzo, Giannini and Miers [27] reported that the belief in negative interpretations appeared to be a clinical feature of anxiety disorder. Indeed, Giannini and Loscalzo [26] argued that such a belief indicated the need for preventive intervention. In support of this, our results showed that IBM reduced negative beliefs and anxiety. In the context of self-relevant imaginations during our modification task, the priority of negative interpretations was apt to lose contact with negative biases and self-images, thus gradually convincing individuals to believe in positivity. Negative thought intrusions can also be reduced during this process [39]. Thus, it is conceivable that manipulating beliefs surrounding negative interpretations is a crucial step towards alleviating anxiety.

Thanks to its positive influences on the interpretation bias, the IBM app effectively reduced anxiety; this effect persisted for at least one month. Some advantages of the IBM app also contributed to anxiety reduction. First, the IBM app adopted the AS-paradigm, which may be the most effective modification task for shifting interpretations [31]. Further, the training materials extensively covered daily ambiguous situations. The combination of the training task and these materials successfully boosted the efficacy of our smartphone-based intervention. Second, a previous study found that approximately 100 trials were the minimum number needed for effect onset each time [16]. Accordingly, we asked participants to complete smartphone-based IBM training twice daily with 50. We also reminded them to complete both sessions each day, meaning that each participant in the training groups completed 56 sessions over 28 days, which is higher than the number implemented in any existing cognitive-bias-modification-related study, to the best of our knowledge. However, we are not implying that having more trials increases the likelihood of a successful IBM intervention. Rather, we emphasise that this was the reason individuals were willing to complete multiple sessions and training trials, as they were increasingly willing to approve this vehicle and the modifying process. Thus, the combination of active willingness and suitable methods are critical factors for interventional success.

Despite these promising results, this study also had two main limitations. First, we only measured anxious feelings using the STAI and not via the Generalized Anxiety Disorder Scale [40] or other measurements that target anxiety symptoms. A single anxiety measurement partly hindered our ability to evaluate whether the smartphone-based IBM app could broadly be used in the clinical context. However, the mean anxiety scores at baseline were higher than those reported in other studies [16,41,42], and the STAI and GAD-7 scores were positively related [43]. Thus, the IBM app demonstrates great potential as a self-supporting tool for clinically diagnosed anxious persons. Second, a previous study suggested that IBM may influence multiple possible mediators to reduce anxiety, such as reappraisal [44]. For example, individuals with anxiety disorder habitually use less cognitive reappraisals, an adaptive emotion-regulation strategy negatively related to anxiety [45]. The reappraisal process reconstructs an emotion-eliciting situation neutrally or positively to alter its emotional impact [30]. Therefore, reappraisal and the practice of making positive interpretations possibly share the same cognitive process, meaning that IBM may reduce anxiety through its implications for emotion-regulation ability. Moreover, cognitive biases share an interactive relationship, meaning that interpretation biases likely exert influences on attention bias [9] or memory bias [24,36], thus jointly contributing to changes in anxiety. Therefore, this study was also limited due to our lack of assessments for other cognitive biases or possible mediators, which may impose more cognitive loads on participants. These limitations also highlight important areas of exploration in future research.

This study demonstrated the effectiveness of delivering IBM via smartphone. While two preliminary studies provided participants with website links to engage in smartphone-conducted IBM training [16,18], both were essentially similar to previous laboratory-based studies. Moreover, one failed to reduce threat biases [18]. Thus, the smartphone-based IBM app used in this study demonstrated significant improvements in effectiveness and feasibility. Another major advantage of this study was the extremely low dropout rate, which provided additional information about dynamic changes in the interpretation bias and anxiety. We attribute this low attrition rate to both the great patience the experimenters exhibited by continually reminding participants to complete their training and the robust training materials, which helped the experiment seem less tedious. We also believe this was critical for increasing user engagement.

## 5. Conclusions

This study demonstrated that a smartphone-based IBM app could improve interpretation bias and anxiety levels. We should also mention that our IBM app was a standalone product and could not receive continual updates. In this regard, we propose the need for an IBM app that can be updated and can target different types of anxiety and relevant symptoms. Meanwhile, additional research is needed to determine which situations and word fragments will maximise the interventional effects. Our results also showed that changes in the number of negative interpretations played a mediating role in anxiety reduction. Thus, future studies may benefit by providing more positive interpretations under each ambiguous situation via word fragments. Worldwide group discussions conducted through Internet communities may also lead individuals to think about more possible positive interpretations. Future studies could also design intervention tasks that change reaction times and/or attitudes towards different interpretations. For example, this may include a new intervention app that requires participants to choose positive interpretations as quickly as possible among many other options. An implicit association test (IAT) may be another feasible task in the smartphone-based IBM; therein, positive interpretations and positive words can be mapped to the same response key, while threatening interpretations and negative words can be mapped to an opposing key. Such associations would largely influence negative information processing in anxious individuals. Broadly speaking, this study establishes a new path for transferring IBM to mobile phones while providing important insights for future clinical interventions. These efforts may further protect individuals from various adversities and gradually facilitate positive changes in related brain areas.

## Figures and Tables

**Table 1:** Participant demographics [Mean (S.D.)].

	Positive Training (*n* = 34)	50-50 (*n* = 31)	NTC (*n* = 29)	F/χ2	*p*-Value
Age (years)	22.4 (2.09)	21.8 (1.92)	22.9 (1.33)	2.68	0.07
Gender (male%)	24%	23%	24%	0.02	0.99
Education (years)	14.9 (1.84)	14.5 (1.65)	15.4 (1.08)	2.26	0.11
Anxiety scores	57.6 (6.13)	58 (5.67)	57.1 (4.92)	0.16	0.85

**Table 2:** The results of follow-up assessment (repeated measures ANOVAs).

Repeated Measures ANOVAs	Main Effect (*p*-Value)			Interaction (*p*-Value)
	Time	Group		Time × Group
Interpretation Bias				
Benign endorsement	0.446	<0.001	Positive > 50-50; Positive > No-training control; 50-50 > No-training control	0.775
Threatening endorsement	0.449	<0.001	Positive < 50-50; Positive < No-training control	0.265
Number	0.459	<0.001	Positive < 50-50; Positive < No-training control	0.375
Rank	0.616	<0.001	Positive > 50-50; Positive > No-training control	0.421
Belief	0.899	<0.001	Positive < 50-50; Positive < No-training control	0.116
Anxiety				
State anxiety	0.028	<0.001	Positive < 50-50; Positive < No-training control; post-test < follow-up	0.619
Trait anxiety	0.116	<0.001	Positive < 50-50; Positive < No-training control	0.704

**Table 3:** The result of the mediation analysis.

The Group Assignment to Mediators				
	Coeff	Se	t	*p*
Benign interpretation Endorsement	−0.124	0.021	5.863	<0.001
Threatening interpretation Endorsement	0.061	0.233	2.6	0.011
Negative rank Interpretations	−0.207	0.463	−4.46	<0.001
Belief about negative interpretations	0.36	0.203	1.772	0.08
Number of negative interpretations	1.655	0.519	3.187	0.002
Direct effects of mediators on anxiety				
	Coeff	Se	t	*p*
Benign interpretation Endorsement	5.085	5.768	0.882	0.3804
Threatening interpretation Endorsement	−2.712	5.668	−0.479	0.634
Negative rank Interpretations	−2.491	2.867	−0.869	0.3872
Belief about negative interpretations	1.151	0.643	1.79	0.077
Number of negative interpretations	0.484	1.502	3.125	<0.001
Total effect of the group assignment on anxiety				
	Effect	Se	t	*p*
	5.63	1.2	4.7	<0.001
Direct effect of the group assignment on anxiety				
	Effect	Se	t	*p*
	4.696	1.502	3.125	0.002
Indirect effect through proposed mediators				
	Effect	BootSe	LLCI	ULCI
Benign interpretation Endorsement	−0.632	0.801	−2.227	0.976
Threatening interpretation Endorsement	−0.164	0.317	−0.792	0.513
Negative rank Interpretations	0.514	0.682	−0.88	1.852
Belief about negative interpretations	0.414	0.319	−0.784	1.162
Number of negative interpretations	0.802	0.481	0.039	1.9

## Data Availability

All data included in this study are available upon request by contacting the corresponding author.
